# Exosomal miR‐1470 is a diagnostic biomarker and promotes cell proliferation and metastasis in colorectal cancer

**DOI:** 10.1002/cam4.7117

**Published:** 2024-03-28

**Authors:** Yawen Wu, Jie Zhang, Fanfeng Lin, Yajing Zhao, Baibing Zheng, Na Zhou, Zhe Zhang, Guanghao Li, Li Xie

**Affiliations:** ^1^ Shandong Provincial Key Laboratory of Radiation Oncology, Cancer Research Center Shandong Cancer Hospital and Institute, Shandong First Medical University and Shandong Academy of Medical Sciences Jinan Shandong China; ^2^ Department of Clinical Laboratory Shandong Cancer Hospital and Institute, Shandong First Medical University and Shandong Academy of Medical Sciences Jinan Shandong China; ^3^ Department of Laboratory Key Laboratory of Cancer Prevention and Therapy, Department of Laboratory, National Clinical Research Center for Cancer, Tianjin's Clinical Research Center for Cancer, Tianjin Medical University Cancer Institute and Hospital Tianjin China; ^4^ Blood Group Reference Laboratory, Shandong Blood Center Jinan Shandong China

**Keywords:** colorectal cancer, diagnostic biomarkers, early detection, exosomes, miR‐1470

## Abstract

**Background:**

In recent years,the lack of specific markers for the diagnosis of colorectal cancer has led to an upward trend in both morbidity and mortality from this condition. There is an urgent need to identify molecular biomarkers that contribute to early cancer detection. This study aimed to identify specific exosomal microRNAs that hold potential as diagnostic biomarkers for CRC.

**Methods:**

We screened for differentially expressed miRNAs using the CRC exosome dataset GSE39833. To validate the results in the public database, we collected serum from 168 CRC patients and 168 healthy volunteers. The expression levels of exosomal miR‐1470 in healthy volunteers and CRC patients were analyzed using qRT‐PCR. To evaluate the diagnostic potential of the selected miR‐1470 in distinguishing CRC patients from healthy controls, we analyzed its receiver operating characteristic curve. To explore the biological functions of miR‐1470 in CRC cell lines, we detected the miR‐1470's ability to regulate the growth and metastasis of CRC cells by CCK8, transwell and other assays after transfection of miR‐1470 in SW480, HCT‐116 cells.

**Results:**

Exosomal miR‐1470 exhibited significant up‐regulation in CRC patients compared to healthy volunteers. The ROC curve analysis revealed an area under the curve (AUC) of 0.74 (95% confidence interval: 0.6876–0.7920) for exosomal miR‐1470, indicating its potential as a diagnostic biomarker. Furthermore, the expression level of miR‐1470 in CRC patients showed correlations with age, metastasis, and HDL content. We overexpressed miR‐1470 in CRC cell lines. CCK8 proliferation assay showed that miR‐1470 promoted the proliferation ability of SW480 and HCT‐116 cells. Transwell assay showed that miR‐1470 promoted the migration and invasion ability of SW480 and HCT‐116 cells.

**Conclusion:**

This suggested that non‐invasive diagnosis of CRC is possible by detecting the level of miR‐1470 in exosomes, which has important implications for early detection and treatment of this disease.

## 1 INTRODUCTION

Colorectal cancer (CRC) is a widespread cancer of the gastrointestinal tract, with significant morbidity and mortality rates. In terms of global cancer incidence, CRC comes in third place.^1^ Epidemiologic studies have provided valuable insights into the complex factors that contribute to the development of CRC. It is increasingly evident that the disease is influenced by a synergistic combination of various environmental, lifestyle, and genetic factors.^2^ It is noteworthy that patients with early‐stage CRC who also have obesity and metabolic syndrome (MetSyn) tend to have lower survival rates. MetSyn is a cluster of conditions that include obesity, high blood pressure, high blood sugar, and abnormal lipid levels. The presence of MetSyn in CRC patients may contribute to more aggressive tumor behavior and poorer treatment outcomes.^3^ Furthermore, liver metastasis in CRC is a significant concern, it is often associated with advanced stages of the disease and is a leading cause of death in these patients.^4^ Lack of early diagnosis of CRC, extensive metastasis, and high tolerance to chemotherapy/radiotherapy contribute to the high mortality rate of CRC. Colonoscopy is currently the reference method for CRC screening,^5^ but mass screening efforts are not justified due to the high cost of colonoscopy and the risk of intestinal perforation during colonoscopy as well as the risk of bleeding after colonoscopy. Currently, carcinoembryonic antigen (CEA) and carbohydrate antigen 19‐9 (CA19‐9) are commonly used as tumor markers for preoperative testing in patients with non‐metastatic CRC. However, it is true that CEA and CA19‐9 do not have high sensitivity and specificity, meaning that they may not be able to accurately detect all cases of CRC or distinguish it from other conditions. This limitation can lead to false‐negative or false‐positive results, potentially affecting the diagnosis and treatment decisions. Therefore, there is an urgent need for novel non‐invasive biomarkers with high sensitivity and specificity for early detection of CRC.

Exosomes are small membrane‐bound vesicles that are actively secreted by various living cells, including cancer cells. They range in size from 50 to 150 nm in diameter, making them smaller than most other types of extracellular vesicles.^6^ Exosomes have been found in plasma, urine, semen, saliva, bronchial fluid, cerebrospinal fluid, serum, lymph, bile, and gastric acid.^7^ Exosomes contain a variety of biologically active substances, and the Exocarta database reveals the composition of exosomes, which contain RNA, proteins, lipids, and microRNAs, among others; the active substances in exosomes can be transferred to receptor cells and perform their functions.^8^ Exosomes are thought to be involved in the development and progression of cancer, and many studies have reported that tumor‐derived exosomes can be involved in intercellular communication and tumor development.^9^

miRNAs are small 21–23 nt non‐coding RNAs that regulate gene expression in eukaryotic cells mainly by binding to the 3′UTR of the target mRNA at the post‐transcriptional level either completely or partially complementary to the 3′UTR of the target mRNA, thus regulating gene expression in eukaryotic cells.^10^ miRNAs are considered to be the most abundant and critical biomolecules in exosomes and play an important regulatory role in tumorigenesis and progression. Exosomes are characterized by a lipid bilayer membrane, which stabilizes the internal components and prevents degradation by certain enzymes.^11^ The components in exosomes change accordingly to the type and physiological state of the cell.^12^ Therefore, this provides a good theoretical basis for the use of exosomal miRNAs as tumor markers. More studies have elucidated circulating exosomal miRNA as a diagnostic biomarker for CRC patients using high‐throughput sequencing. Zhao et al. found that serum exosomal miR‐99b‐5p and miR‐150‐5p levels in CRC patients were significantly lower than those in healthy donors (HD) and benign diseases; exosomal miR‐99b‐5p and miR‐150‐5p expression levels in patients with early stage CRC were significantly lower than those in HD; and exosomal miR‐99b‐5p and miR‐150‐5p expression levels were significantly higher in CRC patients after surgery. This study demonstrates that serum exosomal miRNA is expected to be a sensitive, specific, and noninvasive biomarker for the diagnosis of CRC.^13^ Thus, exosomal miRNA is a promising non‐invasive biomarker for diagnosing CRC and determining whether metastasis has occurred.

Exosomal miR‐1470 is significantly up‐regulated in CRC patients compared to healthy volunteers. The ROC curve analysis revealed an AUC of 0.7398, indicating that exosomal miR‐1470 has moderate accuracy in distinguishing CRC patients from healthy individuals. This finding suggested that exosomal miR‐1470 may have diagnostic potential as a biomarker for CRC. Moreover, the expression level of miR‐1470 in CRC patients is correlated with age, metastasis, and high‐density lipoprotein (HDL) content. These correlations suggested that miR‐1470 may be associated with the clinical characteristics of CRC patients and could potentially serve as a prognostic or predictive marker. In vitro cell experiments, miR‐1470 was found to promote the growth and metastasis of CRC cells. This suggested that miR‐1470 may play a functional role in the development and progression of CRC.

## 2 MATERIALS AND METHODS

### 2.1 Data acquisition and processing

The GSE39833^14^ dataset was utilized to screen for differentially expressed exosomal miRNAs in CRC patients and healthy individuals using the screening criteria of fold change FC >1.5 and *p* < 0.05. Expression of miR‐1470 in CRC tissues and serum was obtained using the GSE108153,^15^ GSE124158,^16^ GSE106817,^17^ GSE59856,^18^ GSE113486^19^ and GSE112264^20^ datasets.

### 2.2 Patients and healthy donors

Serum samples from 168 CRC patients and 168 HD used in this study were collected from January to October 2021 at Shandong Tumor Hospital and stored at −80°C. Serum is produced by the natural coagulation of blood in red‐capped blood collection tubes that do not contain anticoagulants or procoagulants. Requirements for inclusion in the health examination specimen include the absence of benign and malignant tumors, nodules, colon polyps, and any other endocrine, immune, or metabolic disorders. CRC patients were diagnosed with CRC by a combination of clinical, pathological, and radiological diagnostic methods, and tumor stage was determined according to the American Joint Committee on Cancer (AJCC) 8th edition, and the requirements for enrollment were the absence of a primary tumor elsewhere, the absence of antitumor therapy, and the absence of other endocrine, immune, or metabolic disorders. The clinical characteristics of all patients are shown in Table 1.

**TABLE 1:** Characteristics of CRC patients for differentially expressed serum exosomal miR‐1470.

Characteristic	Cases (*n*)	Mean ± SD	*p*‐value
Gender			
Male	104	−1.020 ± 1.242	0.2639
Female	64	−1.237 ± 1.178	
Age (years)			
<61	90	−0.8560 ± 1.107	0.0143*
≥61	78	−1.316 ± 1.277	
AlcohoL			
Yes	38	−1.190 ± 1.109	0.6164
No	130	−1.077 ± 1.253	
Hypertension			
Yes	53	−1.091 ± 1.177	0.9324
No	115	−1.108 ± 1.243	
Diabetes			
Yes	32	−1.329 ± 1.370	0.2432
No	136	−1.049 ± 1.180	
Tumor position			
Colon	68	−1.178 ± 1.310	0.5124
Rectum	100	−1.051 ± 1.158	
Lymph node metastasis			
Yes	72	−1.341 ± 1.098	0.0019**
No	75	−0.7309 ± 1.237	
Unknown	21	−1.610 ± 1.217	
Distant metastasis			
Yes	40	−1.286 ± 1.026	0.1377
No	115	−0.9633 ± 1.226	
Unknown	13	−1.769 ± 1.488	
Stage			
0–II	64	−0.8721 ± 1.164	0.0068*
III–IV	74	−1.391 ± 1.054	
Unknown	30	−0.8810 ± 1.563	

### 2.3 Exosome extraction

Collected 1 mL serum samples are centrifuged at 12,000*g* at 4°C to remove erythrocytes and debris, centrifuged at 10,000*g* for 30 min at 4°C to remove microvesicles from the serum, and then centrifuged at 100,000*g* ultracentrifugation for 2 h at 4°C to obtain exosomes.

### 2.4 qNano

Exosome samples extracted by ultracentrifugation were resuspended in 100 μL of PBS and then analyzed for exosome particle size using a qNano instrument (Izon Sciences Ltd.) and Izon Control Suite v.3.3.2.2001 software.

### 2.5 Transmission electron microscopy assay

The collected exosomes were first fixed with glutaraldehyde, and then the exosome solution was added dropwise to the copper mesh and adsorbed for about 10 min at room temperature, and the excess liquid was carefully absorbed with filter paper. Then 10 μL of 2% phosphotungstic acid solution (pH = 6.5) was added dropwise to the copper mesh, and the exosomes were stained and processed for 2 min at room temperature, the excess staining liquid was carefully absorbed with a filter paper, and the copper mesh was air‐dried at room temperature, and then photographed with an electron microscope (JEM1200EX, Jeol, Japan) for detection.

### 2.6 Cell culture

Human CRC cell lines SW480 and HCT‐116 were purchased from Shanghai Cell Bank. The cells were cultured in 1640 medium containing 10% fetal bovine serum (FBS; Thermo Fisher Scientific, USA) at 37°C in a humidified environment with 5% CO_2_. Overexpression of miR‐1470 mimic (Gemma China) was synthesized and CRC cells were transfected with Lipofectamine 3000 reagent (Invitrogen) according to the manufacturer's instructions.

### 2.7 Western blot analysis

Protein lysate containing 0.5% PMSF was added to CRC cell precipitates (SW480 and HCT‐116 cells) and exosomes obtained by ultracentrifugation and placed on ice for adequate lysis. The above protein extracts were then separated by 10% SDS‐PAGE and transferred to PVDF membranes (Millipore, USA). The membranes were closed with 5% skimmed milk for 3 h at room temperature, incubated with anti‐GM130, HSP70 and CD9 primary antibodies (all 1:500, Cell Signaling Technology, USA) at 4°C overnight, and then incubated with horseradish peroxidase‐conjugated (HRP)‐conjugated secondary antibodies (1:1) for 1 h at room temperature before protein streaks were detected by using ECL blotting assay reagent to develop the protein bands and fix them on x‐ray film.

### 2.8 RNA isolation and quantitative real time PCR

After ultracentrifugation, 500μL of Trizol was added to each exosome sample for further RNA extraction and reverse transcription to cDNA using the miRNA First Strand Reverse Transcription Kit (AG11716). Forty cycles of real‐time quantitative PCR (Roche LightCycler 480 System, Switzerland) were performed on the samples to detect the target gene and internal reference gene (U6). The relative expression level of exosomal miRNAs was calculated as ΔCT = CT^miRNA^‐CT^U6^, with a smaller ΔCT representing a higher concentration of miRNA.

### 2.9 Cell proliferation assay

The transfected CRC cells were inoculated into 96‐well plates (2000 cells per well) and incubated at 37°C. The cells were incubated with CCK8 reagent (sparkjade, China) for 1 h at a fixed time every day, and the absorbance was detected at 450 nm with an enzyme marker.

### 2.10 Cell migration and invasion assay

Transfected CRC cells 6 × 10^4^ were inoculated in the upper lumen of a 0.8 μm Transwell (Corning), and a 25% matrix gel (Corning 0827065) was applied to the Transwell for the invasion assay, and the migrated CRC cells were fixed with formaldehyde for 30 min after 24 h of incubation and stained with 0.1% crystal violet solution. Then they were counted by light microscope and photographed.

### 2.11 Pathway enrichment analysis

To clarify the biological function of hsa‐miR‐1470 in CRC, we first obtained the potential target genes of miR‐1470. We performed differential expression analysis using the TCGA‐CRC cohort and identified mRNAs with FC >2 and FDR <0.05 as differentially expressed genes. Subsequently, we obtained target genes of miR‐1470 using Targetscan (TargetScanHuman 8.0) and miRPathDB online sites (miRPathDB [uni‐sb.de]) and crossed these target genes with differentially expressed genes. Finally we analyzed the crossover genes for GO and KEGG enrichment using the R package clusterprofiler. Pathways with *p* < 0.05 were considered statistically significant.

### 2.12 Statistical analysis

Statistical analyses were performed using SPSS 22.0 (IBM Corp, USA) software, Xiantao online tool (https://www.xiantaozi.com/), and GraphPad Prism version 9.0 (GraphPad Software, San Diego, CA, USA). ROC curves and area under the curve (AUC) were used to detect diagnostic efficiency. Statistical analyses were performed using *t*‐test. Pearson correlation analysis was used to assess the degree of linear relationship between miR‐1470 and clinical indicators in CRC patients. All results were expressed as mean ± SD (standard deviation), and *p* < 0.05 was considered as statistically significant difference.

## 3 RESULTS

### 3.1 Screening for miR‐1470 using the GEO datasets

**FIGURE 1:**
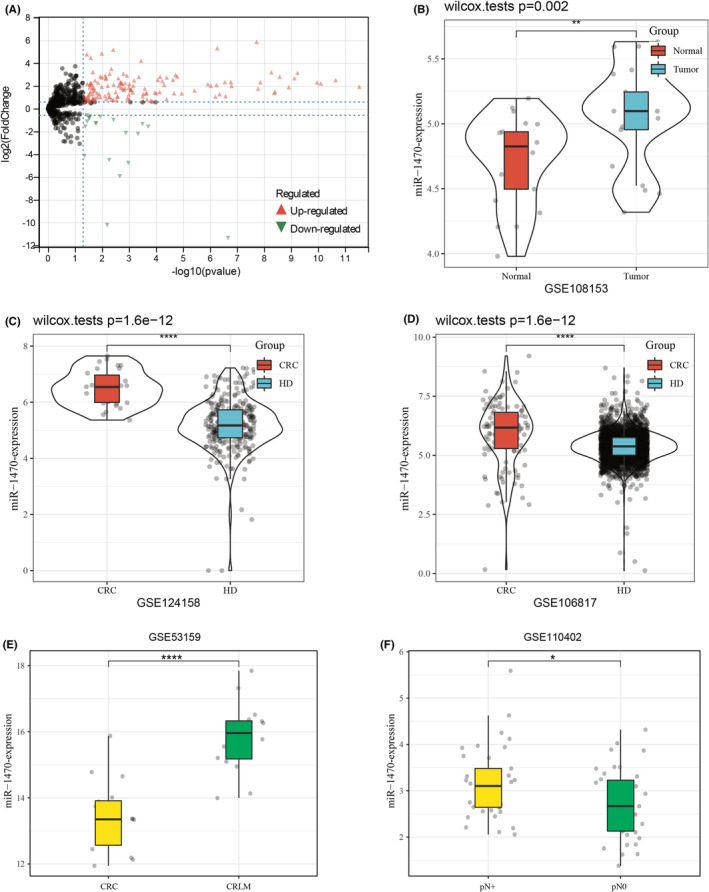
Differentially expressed exosomal miRNAs between CRC and healthy individuals in the GEO database (A) Screening for differentially expressed exosomal miRNAs in CRC patients and healthy individuals using the GSE39833 database Red color indicates up‐regulated miRNAs, while green color indicates down‐regulated miRNAs. (B) The expression level of miR‐1470 in GSE108153 colorectal cancer (CRC) tissue database. (C, D) Expression levels of miR‐1470 in GSE124158 and GSE106817 CRC serum databases. (E, F) Expression levels of miR‐1470 in patients with CRC liver metastasis and CRC patients without liver metastasis in the GEO database. (G) Expression levels of miR‐1470 in CRC patients with lymph node metastasis and CRC patients without lymph node metastasis in the GEO database (**p < 0.01, ****p < 0.0001).

We performed a screen in GSE39833 to investigate differentially expressed exosomal miRNAs in CRC patients and HD. We applied the criteria of fold change FC >1.5 and *p* < 0.05 and identified a total of 75 up‐regulated and 19 down‐regulated exosomal miRNAs (Figure 1A). After conducting a comprehensive literature review, we excluded the miRNAs that had previously been identified as differentially expressed in CRC. Additionally, we removed miRNAs with poor primer specificity and low expression levels. Consequently, our focus narrowed down to specifically target the gene miR‐1470. To verify the expression of miR‐1470 in CRC tissues and serum, we conducted an analysis using the GEO datasets. This allowed us to obtain additional validation for our findings. After analyzing the GEO datasets GSE108153, we observed a significant upregulation of miR‐1470 expression in CRC tissues compared to normal tissues (Figure 1B). Moreover, our analysis of the GEO serum datasets, specifically GSE124158 and GSE106817, revealed a notable increase in miR‐1470 expression in the serum of CRC patients when compared to the serum of HD (Figure 1C,D). Additionally, analysis of miR‐1470 expression levels in several other serum datasets sourced from the GEO (GSE59856, GSE113486, and GSE112264) revealed that miR‐1470 expression levels were significantly higher in CRC patients compared to healthy volunteers (Figure S1). In examining the relationship between miR‐1470 expression levels and distant metastasis and lymph node metastasis using the GEO datasets, we found that miR‐1470 expression levels were higher in CRC patients with liver metastasis than in those without metastasis. In addition, miR‐1470 expression levels were higher in CRC patients with lymph node invasion compared to those without lymph node metastasis (Figure 1E,F).

### 3.2 Identification of isolated exosomes and characterization of exosome miR‐1470

**FIGURE 2:**
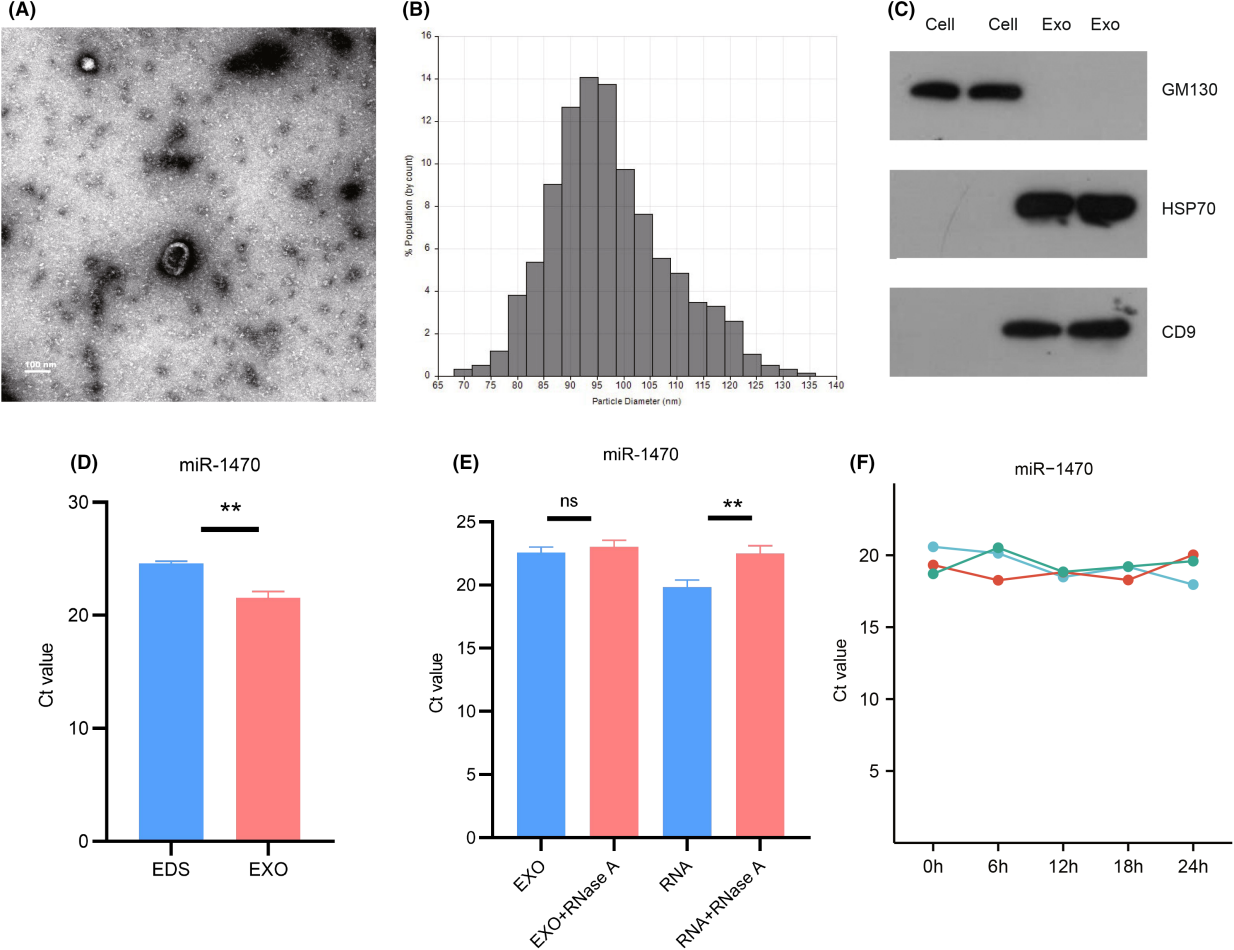
Identification of exosomes and characterization of serum exosome miR‐1470 (A) TEM images showing exosomes isolated from serum of CRC patients. (Scale bar: 100 nm; high voltage (HV) = 80 kV). (B) Plasma exosomes were detected based on the qNano system, and the diameters were mainly distributed between 80 and 120 nm. (C) GM130 and exosomal protein markers: CD9 and HSP70. (D) Expression levels of miR‐1470 in serum exosomes (EXO) and exosome clearance supernatant (EDS). (E) qRT‐PCR analysis of the expression levels of miR‐1470 in the exosomes or isolated nucleic acids treated with RNase A. (F) Expression levels of miR‐1470 in serum exosomes at different incubation times at room temperature (**p < 0.01; ****p < 0.0001. EDS, exosome‐depleted supernatant; EXO, Exosomes; ns; not significant).

Following the ultracentrifugation‐based extraction of the serum, transmission electron microscopy (TEM) analysis was performed, revealing the presence of double‐membrane elliptical microcapsule structures in the extract (Figure 2A). Analysis using qNano revealed that the diameter of the extracted structures predominantly ranged from 70 to 130 nm, aligning with the typical size range associated with exosomes and confirming their basic morphological characteristics (Figure 2B). In the final step, Western blot experiments revealed that CD9 and Hsp70, known markers for exosomes, were enriched in the serum of CRC patients, while their expression was not detected in CRC cells (SW480 and HCT‐116), and GM130 (negative control) was expressed in CRC cells but not in exosomal extracts **(**Figure 2C **)**. In conclusion, the performed experiments, including TEM observation, qNano analysis, and Western blot verification, provide strong evidence for the presence of exosomal structures, the characteristic size distribution, and the enrichment of exosomal markers such as CD9 and Hsp70. Based on these findings, it can be confidently concluded that the extracted material is representative of exosomes. Subsequently, we further confirmed the distribution of miR‐1470 in human serum. RT‐qPCR results showed that the expression level of miR‐1470 in exosomes was higher than that in exosome‐depleted supernatant (EDS) (Figure 2D).

Furthermore, we conducted additional experiments to assess the stability of exosomal miR‐1470. Surprisingly, even after treating the exosomes with RNAse A, an enzyme that degrades RNA, the expression level of exosomal miR‐1470 remained unchanged. This observation suggested that exosomal miR‐1470 is highly stable and resistant to RNA degradation (Figure 2E). Moreover, we incubated the serum exosomes at room temperature for different time periods (0, 6, 12, 18, and 24 h). Remarkably, we observed no significant change in the expression level of exosomal miR‐1470 throughout the incubation period. This finding provided further evidence that exosomes serve as protective carriers, shielding miRNAs from degradation by RNase. Furthermore, it highlighted the exceptional stability of exosomal miRNAs within the exosomes themselves (Figure 2F).

### 3.3 Serum exosomal miR‐1470 as a diagnostic marker in CRC

**FIGURE 3:**
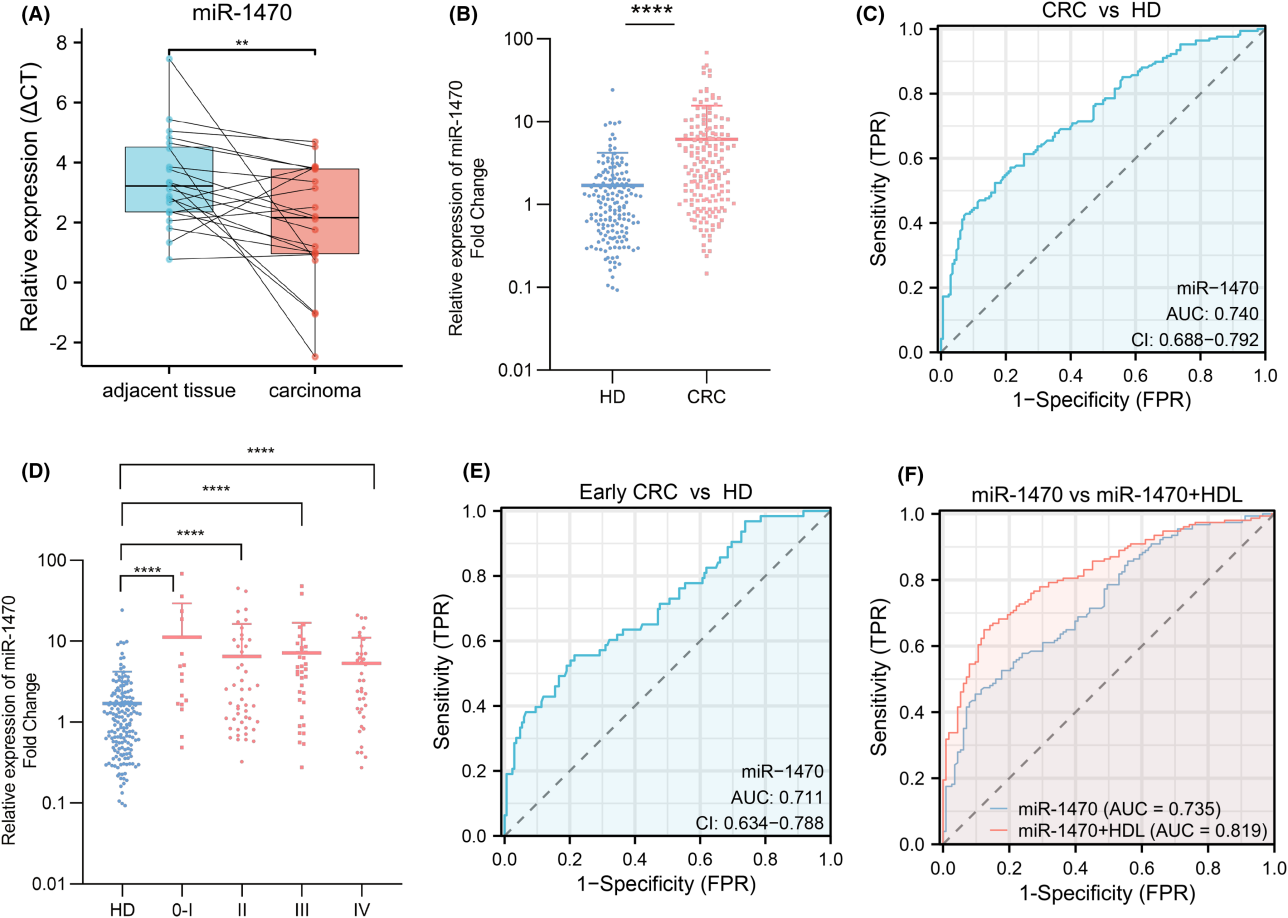
Increased expression of exosomal miR‐1470 in CRC and its use as a CRC diagnostic biomarker (A) miR‐1470 expression levels in colorectal cancer (CRC) tissues and paracancerous tissues. (B) Levels of miR‐1470 in serum exosomes from healthy population and CRC patients. (C) The areas under the curve (AUCs) of serum exosomal miR‐1470 in 168 CRC patients and 168 HD. (D) Levels of miR‐1470 in serum exosomes of healthy people and CRC patients at different stages. (E) The areas under the curve (AUCs) of serum exosomal miR‐1470 in 63 early‐stage CRC patients and 168 HD. (F) The AUC values of miR‐1470 combined with HDL in CRC patients (**p < 0.01, ****p < 0.0001).

To verify the expression of miR‐1470 in CRC tissues and their adjacent paracancerous tissues. A total of 20 pairs of samples were collected and examined for the expression level of miR‐1470. The results revealed a significant up‐regulation of miR‐1470 in CRC tissues when compared to the adjacent paracancerous tissues (Figure 3A). Our findings were consistent with the predicted changes in miR‐1470 expression levels in the GEO dataset, which enhances the reliability of our findings. To investigate non‐invasive specific tumor markers, we further analyzed serum exosomes samples from healthy volunteers and primary CRC patients. The results showed that the expression of miR‐1470 in serum exosomes from CRC patients was increased by about 4.5‐fold (*p* < 0.0001) compared with serum exosomes from healthy volunteers (Figure 3B). The ROC curves demonstrated the performance of exosomal miR‐1470 as a diagnostic marker for CRC. In this case, the AUC of exosomal miR‐1470 was 0.74 (95% CI: 0.6876–0.7920). This suggested that exosomal miR‐1470 has a high discriminatory ability in distinguishing CRC patients from healthy individuals (Figure 3C). We conducted further analysis on the expression levels of serum exosomal miR‐1470 in CRC patients at different stages. The results revealed that the expression levels of exosomal miR‐1470 were significantly higher in stage 0–I CRC patients compared to healthy volunteers (9.5‐fold increase), stage II CRC patients (4.8‐fold increase), stage III CRC patients (5‐fold increase), and stage IV CRC patients (3.6‐fold increase) (*p* < 0.0001) (Figure 3D). To evaluate the diagnostic performance of exosomal miR‐1470 in early‐stage CRC (stage 0–II) patients versus healthy controls, we constructed and analyzed a separate ROC curve. The results showed that exosomal miR‐1470 exhibited good diagnostic efficiency in differentiating early‐stage CRC patients from healthy controls. The calculated AUC was 0.711 (95% CI: 0.634–0.788) (Figure 3E). This finding suggested that the upregulation of miR‐1470 expression was more prominent in the early stages of CRC and might serve as a potential biomarker for early detection of CRC. To investigate the association between the expression level of exosomal miR‐470 and lipid indices in CRC patients, we conducted an analysis of clinical cases. The analysis revealed that a significant majority of CRC patients (88%) exhibited below‐normal levels of HDL. Moreover, our assessment of the relationship between exosomal miR‐1470 expression levels and HDL in CRC patients revealed a substantial rise in the AUC, which reached the value of 0.819 (95% CI: 0.7710–0.8695) (Figure 3F). This increase in the AUC suggested that the combination of exosomal miR‐1470 expression levels and HDL might serve as a valuable diagnostic tool for identifying CRC.

### 3.4 Characteristics of colorectal cancer patients for differentially expressed exosomal miR‐1470

**FIGURE 4:**
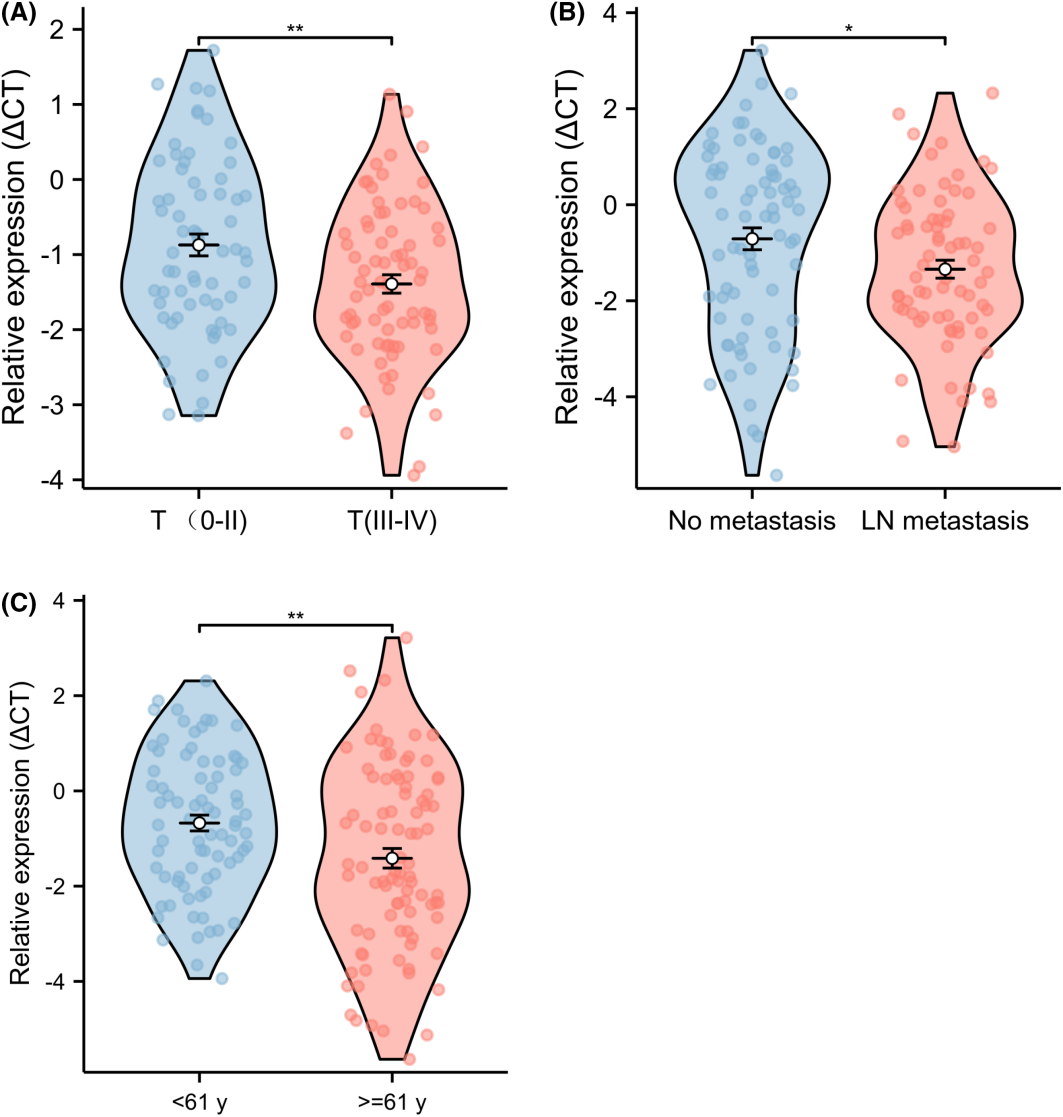
Analysis of serum exosome miR‐1470 and clinical characteristics of CRC patients. (A) Expression levels of exosomal miR‐1470 in T‐stage CRC patients. (B) The expression level of exosomal miR‐1470 in CRC patients with lymphatic metastasis was higher than that in patients without metastasis. (C) The expression level of miR‐1470 in CRC patients over 61 years old was higher than that in CRC patients under 60 years old (*p < 0.05, **p < 0.01, ****p < 0.0001).

In our study, we performed a comprehensive analysis of the clinical characteristics of 168 primary CRC patients. The clinical parameters we assessed included age, gender, smoking history, alcohol consumption, hypertension, diabetes, tumor location, presence of lymph node metastases, distant metastases, and TNM stage. Table 1 summarizes the clinical characteristics of the CRC patients. Our analysis revealed significant associations between miR‐1470 expression level and certain clinical parameters in CRC patients. Specifically, we observed correlations between miR‐1470 expression and age, lymph node metastasis, and tumor stage. The expression of exosomal miR‐1470 was found to be significantly higher in patients with advanced‐stage CRC (stage III–IV) compared to patients with early‐stage CRC (stage 0–II) (Figure 4A). This suggests that miR‐1470 may play a role in the progression and aggressiveness of CRC, with its expression levels potentially increasing as the disease advances. Our analysis revealed that exosomal miR‐1470 expression levels were significantly higher in CRC patients with lymph node metastasis compared to those without lymph node metastasis (Figure 4B). And exosomal miR‐1470 expression levels were found to be higher in CRC patients aged over 61 years compared to CRC patients under 61 years of age (Figure 4C). This suggested that age might influence the expression of exosomal miR‐1470 in CRC patients, with older patients exhibiting higher levels of miR‐1470.

### 3.5 miR‐1470 regulated the proliferation, invasion, and migration of CRC cells

**FIGURE 5:**
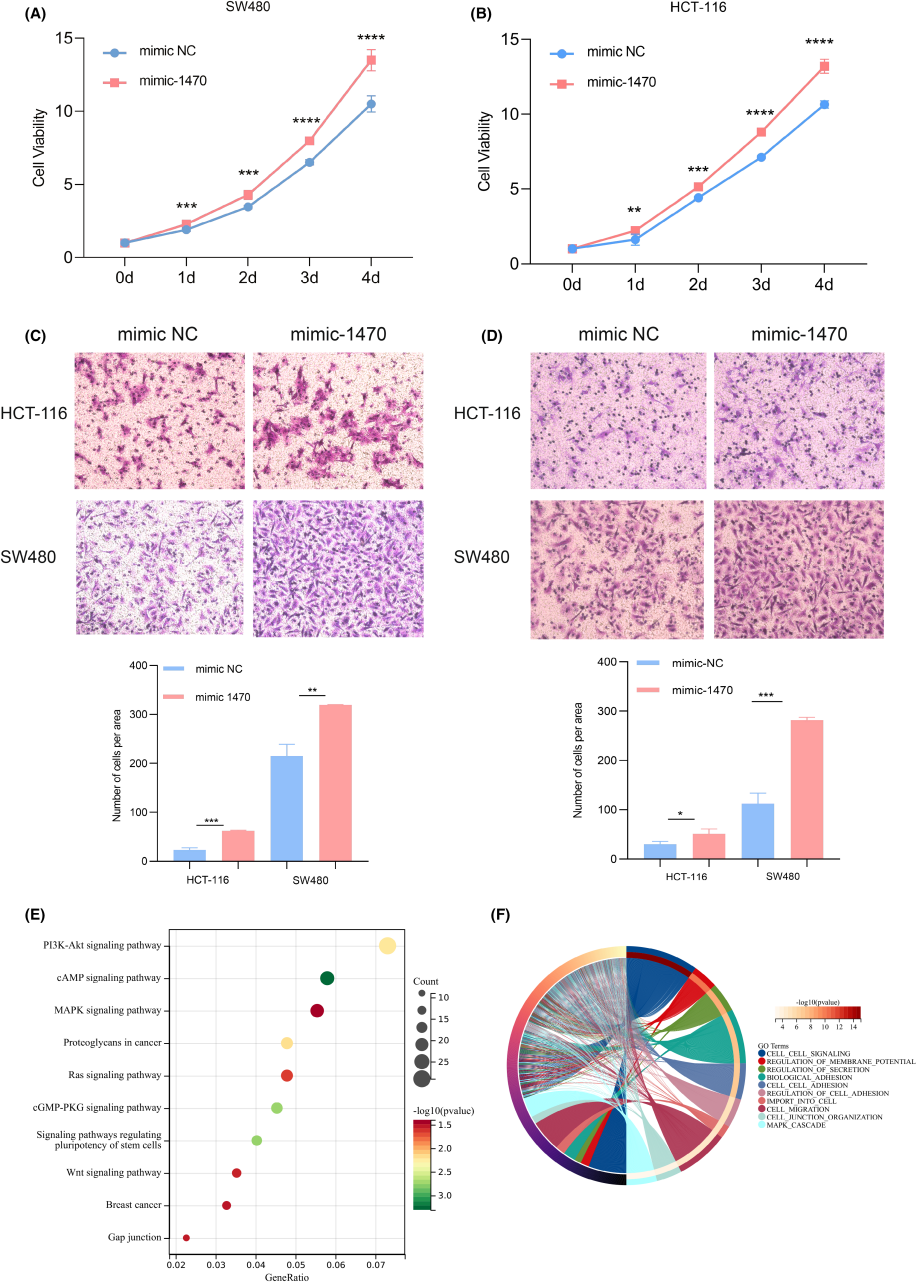
miR‐1470 is involved in regulating the proliferation and migration ability of colorectal cancer CRC cells (A, B) CCk8 experiments illustrated that miR‐1470 promotes the proliferation ability of CRC cells (A) SW480 cells; (B) Hct‐116 cells. (C, D) Transwell experiments illustrated that miR‐1470 promotes migration (C) and invasion (D) of CRC cells ability. (E) Signaling pathways mainly involved in miR‐1470 as shown by KEGG analysis. (F) Biological processes mainly involved in miR‐1470 as shown by GOBP score. (*p < 0.05, **p < 0.01, ***p < 0.001, ****p < 0.0001)

Since miR‐1470 expression is upregulated in early stage CRC, we hypothesized that miR‐1470 may promote CRC progression. After transfecting CRC cells with miR‐1470 mimics, we performed a series of in vitro cellular experiments using CCK‐8 and Transwell assays. The expression efficiency of miR‐1470 in SW480 and HCT‐116 cells was examined by qRT‐PCR (Figure S2A,B). Through CCK‐8 experiments, we observed that the overexpression of miR‐1470 significantly enhanced the proliferative ability of both SW480 (Figure 5A) and HCT‐116 cells (Figure 5B). Furthermore, the overexpression of miR‐1470 significantly promoted the migration ability of CRC cells (Figure 5C) and their invasion ability (Figure 5D) compared to the control group. After conducting our statistical analysis, we discovered that the overexpression of miR‐1470 in CRC cells resulted in a significant enhancement of migration ability, with an approximate 2.5‐fold increase, as well as invasion ability, with an approximate 1.65‐fold increase, in the Transwell assay after a 24‐h. The findings strongly support the hypothesis that miR‐1470 plays a pivotal role in promoting the progression and aggressiveness of CRC. To explore the biological function and potential molecular mechanisms of miR‐1470 in CRC, we explored the differentially expressed mRNAs in the TCGA‐CRC cohort using Xiantao Academic and crossed them with miRPathDB and TargetScan predicted target genes (Figure S2C). Subsequently, we performed pathway enrichment analysis using target genes with these specific potential regulatory relationships. KEGG enrichment analysis revealed that the target genes of miR‐1470 are mainly involved in signaling pathways such as PI3K‐AKT signaling pathway, cAMP signaling pathway, and MAPK signaling pathway (Figure 5E). Additionally, the GOBP analysis (Figure 5F) suggested that the target genes of miR‐1470 are mainly involved in processes such as cell signaling, regulation of membrane potential, regulation of secretion, biological adhesion, cell adhesion, and import into cell, cell migration, cell junction organization, and MAPK cascade signaling pathways.

## 4 DISCUSSION

The absence of early, specific, and noninvasive diagnostic markers stands as a significant contributing factor to the heightened mortality rate of CRC. Therefore, the main objective of this study was to screen specific exosomal miRNAs as biomarkers for the diagnosis of CRC.

miR‐1470 is a short (20–24 nt) non‐coding miRNA located in chromatin 19p13.12, which is involved in the post‐transcriptional regulation of multicellular gene expression. The current study found that miR‐1470 expression levels in exosomes of mesenchymal stem cells (MSCs) increased the proportion of CD4^+^CD25^+^FOXP3^+^ cells in asthma patients.^21^ In addition, miR‐1470 was found to regulate the progression of diabetic retinopathy.^22^ In addition to this, several studies have reported that miR‐1470 may be involved in tumor progression. For example, LU et al. found that miR‐1470 could promote cancer progression by targeting ALX4 in hepatocellular carcinoma (HCC).^23^ In addition, other research groups have found that overexpression of miR‐1470 in esophageal squamous cell carcinoma cells promotes proliferation and migration and inhibits cellular senescence.^24^ However, no studies on the diagnosis and mechanism of action of miR‐1470 in CRC have been reported to date. Our study suggested that exosomal miR‐1470 might serve as a specific diagnostic marker for CRC and promoted the progression of CRC. We expect that this study will provide new methods and approaches for early diagnosis of CRC.

Analysis of the GEO dataset revealed that miR‐1470 was highly expressed in patients with metastatic CRC and was consistent with the clinical characteristics of the patients, suggesting that it might be involved in promoting the invasive behavior of the cancer cells and facilitating the spreading of the tumor cells to the lymph nodes and to the distant sites, which was further illustrated by in vitro experiments. In addition, we found that the expression level of miR‐1470 was correlated with the age of CRC patients, which may imply that miR‐1470 is associated with changes in the immune system function, metabolic changes, or other age‐related biological processes in CRC patients, and also provides a new direction for further research.

The observation that most CRC patients had lower than normal levels of HDL is an interesting finding. HDL is commonly referred to as “good cholesterol” due to its beneficial effects on cardiovascular health. Low HDL levels in CRC patients may be caused by malnutrition in tumor patients or by the tumor affecting lipid metabolism, resulting in lower HDL levels. Literature reports have shown that serum HDL levels correlate with tumor size and stage of CRC, which further suggested that HDL levels may be associated with the severity and prognosis of CRC.^25^ In addition, a team investigation also found that elevated TG/HDL‐C ratio was also associated with an increased risk of CRC in adults in northern China.^26^ Assessing HDL levels alone in patients with CRC does not necessarily provide diagnostic or predictive value. However, when HDL levels in patients with CRC were analyzed in conjunction with exosome miR‐1470, a significantly enhanced diagnostic effect was observed. This combined analysis allows for a more comprehensive assessment of a patient's risk of CRC and provides us with new indicators and methods for diagnosing and predicting CRC risk.

Finally, in our GO analysis can reveal the specific functions that miR‐1470 is involved in, such as cell signaling, regulation of membrane potential, regulation of secretion, cell migration, cell junction organization and so on. KEGG analysis reveal the miR‐1470 may be associated with a variety of pathways, such as PI3K‐AKT, cAMP, and MAPK. The PI3K‐AKT pathway plays a crucial role in the development of CRC. This pathway inhibits apoptosis and promotes proliferation and growth of CRC cells, contributing to tumor development and metastasis.^27^ In CRC, aberrant activation or dysregulation of the cAMP pathway is associated with tumorigenesis, progression, and drug resistance.^28^ The MAPK pathway is a signaling pathway involved in fundamental biological processes including cell survival, proliferation, differentiation, and metastasis.^29^ These pathways are closely related to the normal physiological processes of cells, and the abnormal expression of miR‐1470 may lead to the disruption of these functions, thus promoting tumorigenesis and development.

Nonetheless, this study has several limitations. First, the study sample size was limited to samples collected solely from Shandong Cancer Hospital, potentially compromising the statistical robustness due to the small overall sample size and geographical restriction.

## 5 CONCLUSION

The exosomal miR‐1470 holds promise as a potential biomarker for CRC, offering a valuable avenue for the advancement of non‐invasive diagnostic tools.

## AUTHOR CONTRIBUTIONS

**Li Xie:** Funding acquisition (equal). **Yawen Wu:** Writing – original draft (equal). **Jie Zhang:** Data curation (equal). **Fanfeng Lin:** Formal analysis (equal). **Yajing Zhao:** Investigation (equal). **Baibing Zheng:** Software (equal). **Na Zhou:** Validation (equal). **Zhe Zhang:** Validation (equal). **Guanghao Li:** Writing – review and editing (equal).

## FUNDING INFORMATION

This work was supported by the National Natural Science Foundation of China (81773237), Shandong Province key research and development plan (2021LCZX04), Shandong Provincial Natural Science Foundation (ZR2020LZL017), China Postdoctoral Science Foundation (2023M732126).

## CONFLICT OF INTEREST STATEMENT

The authors declare no potential conflicts of interest.

## ETHICS STATEMENT

The study was conducted in accordance with the principles of the Declaration of Helsinki, and the study protocol was approved by the Ethics Committee of Shandong Cancer Hospital.

## Supporting information


**Figure S1:** The expression levels of miR‐1470 in other GEO serum databases (A) GSE59856, (B) GSE113486, (C) GSE112264 (D) Diagnostic efficacy of miR‐1470, CEA and miR‐1470 combined with CEA as diagnostic of CRC. (E) Diagnostic efficacy of miR‐1470, CEA and miR‐1470 combined with CEA as diagnostic of Early CRC. (****p < 0.0001).

**Figure S2:** The expression efficiency of miR‐1470 in SW480 (A) and HCT‐116 (B) cells was detected by qRT‐PCR. (C) The Venn diagram illustrates the predicted miR‐1470 target genes obtained from TCGA‐CRC, miRPathDB, and TargetScan. (***p < 0.001).

## Data Availability

The original contributions presented in the study are included in the article/Supplementary Material, further inquiries can be directed to the corresponding author.
