# Supplementary figures and images for: Exosomal miR‐1470 is a diagnostic biomarker and promotes cell proliferation and metastasis in colorectal cancer
Source: Cancer Med. 2024 Mar 28;13(7):e7117. doi: 10.1002/cam4.7117 (PMC10974715; doi:10.1002/cam4.7117)

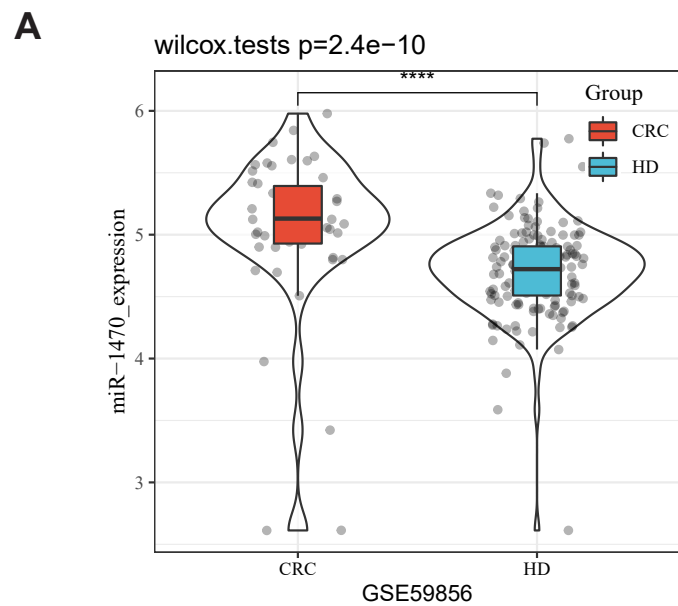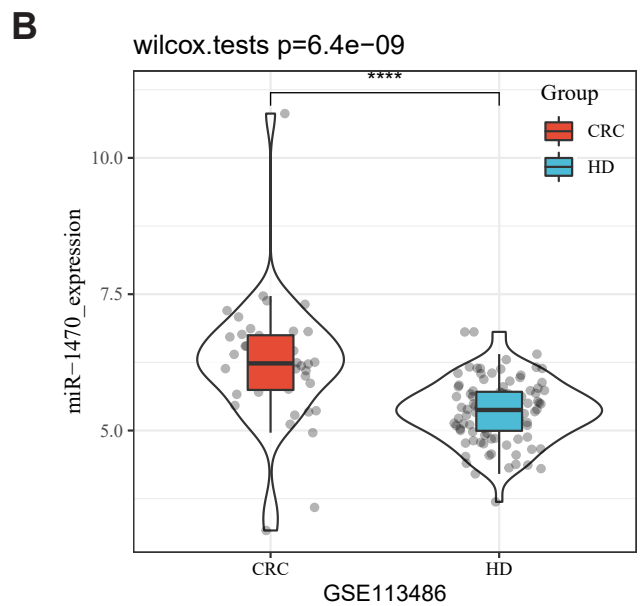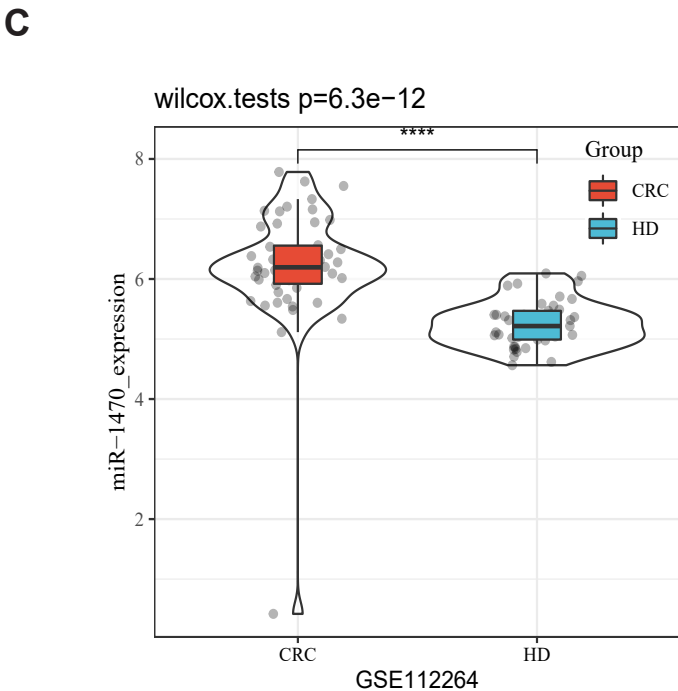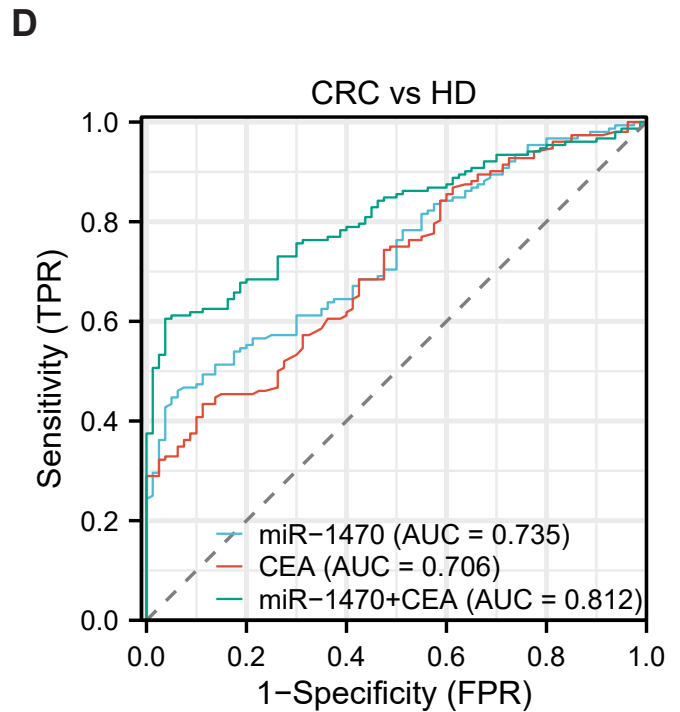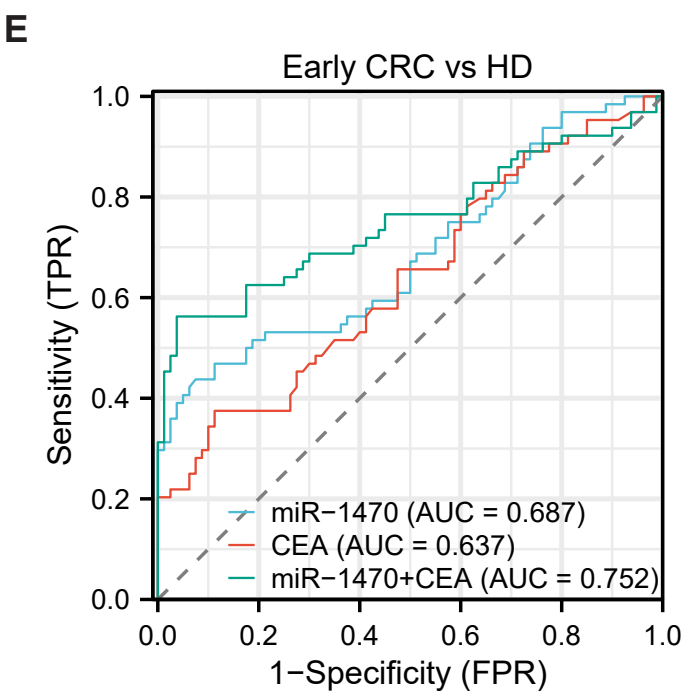

Supplement: Supplementary file 1 — Figure S1: The expression levels of miR‐1470 in other GEO serum databases (A) GSE59856, (B) GSE113486, (C) GSE112264 (D) Diagnostic efficacy of miR‐1470, CEA and miR‐1470 combined with CEA as diagnostic of CRC. (E) Diagnostic efficacy of miR‐1470, CEA and miR‐1470 combined with CEA as diagnostic of Early CRC. (****p < 0.0001). [file CAM4-13-e7117-s002.pdf]

**A**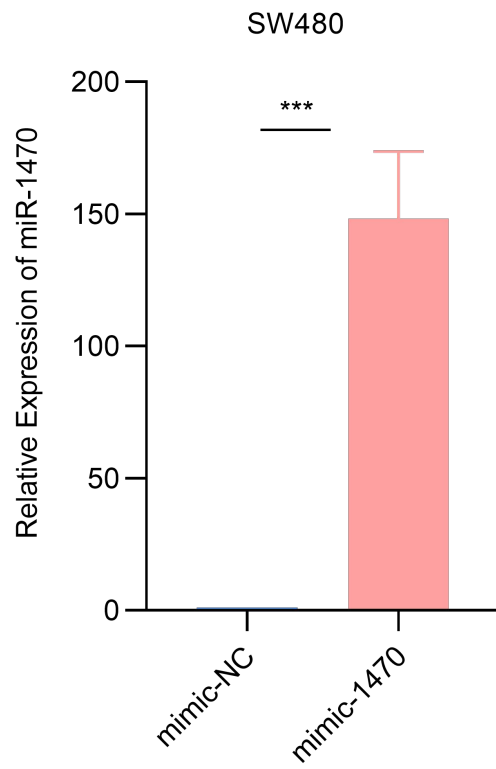**B**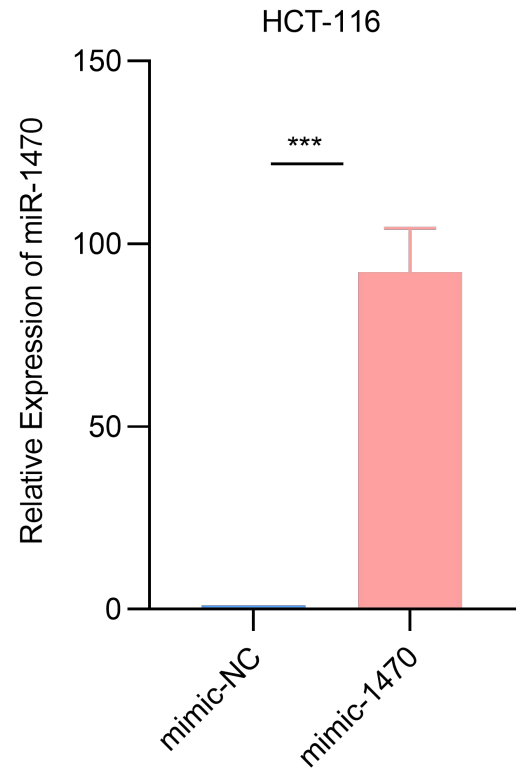**C**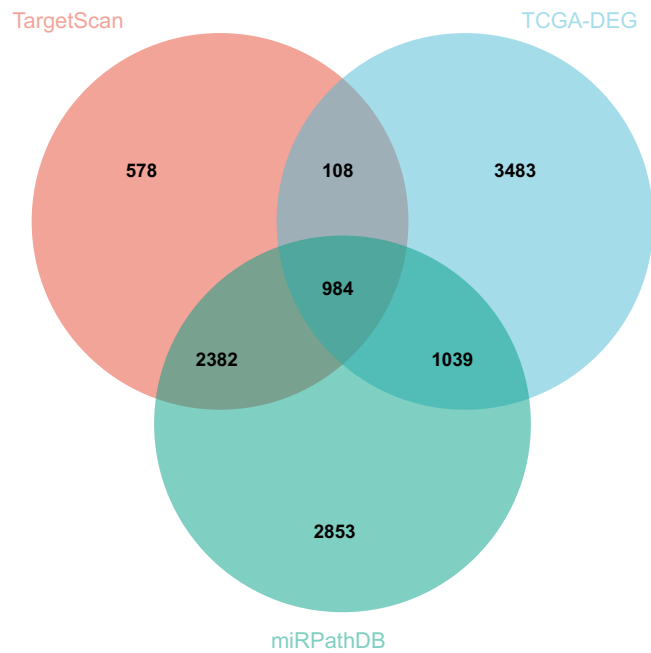

Supplement: Supplementary file 2 — Figure S2: The expression efficiency of miR‐1470 in SW480 (A) and HCT‐116 (B) cells was detected by qRT‐PCR. (C) The Venn diagram illustrates the predicted miR‐1470 target genes obtained from TCGA‐CRC, miRPathDB, and TargetScan. (***p < 0.001). [file CAM4-13-e7117-s001.pdf]
